# How to Evaluate Theory-Based Hypotheses in Meta-Analysis Using an AIC-Type Criterion

**DOI:** 10.3390/e24111525

**Published:** 2022-10-25

**Authors:** Rebecca M. Kuiper

**Affiliations:** Department of Methodology and Statistics, Utrecht University, Padualaan 14, 3584 CH Utrecht, The Netherlands; r.m.kuiper@uu.nl

**Keywords:** confirmatory research, inequality-constraints, information theoretic criteria, informative hypothesis, meta-analysis, model selection, multiple studies, order-restricted hypothesis, theory-based hypothesis

## Abstract

Meta-analysis techniques allow researchers to aggregate effect sizes—like standardized mean difference(s), correlation(s), or odds ratio(s)—of different studies. This leads to overall effect-size estimates and their confidence intervals. Additionally, researchers can aim for theory development or theory evaluation. That is, researchers may not only be interested in these overall estimates but also in a specific ordering or size of them, which then reflects a theory. Researchers may have expectations regarding the ordering of standardized mean differences or about the (ranges of) sizes of an odds ratio or Hedges’ *g*. Such theory-based hypotheses most probably contain inequality constraints and can be evaluated with the Akaike’s information criterion type (i.e., AIC-type) confirmatory model selection criterion called generalized order-restricted information criterion (GORICA). This paper introduces and illustrates how the GORICA can be applied to meta-analyzed estimates. Additionally, it compares the use of the GORICA to that of classical null hypothesis testing and the AIC, that is, the use of theory-based hypotheses versus null hypotheses. By using the GORICA, researchers from all types of fields (e.g., psychology, sociology, political science, biomedical science, and medicine) can quantify the support for theory-based hypotheses specified a priori. This leads to increased statistical power, because of (i) the use of theory-based hypotheses (cf. one-sided vs. two-sided testing) and (ii) the use of meta-analyzed results (that are based on multiple studies which increase the combined sample size). The quantification of support and the power increase aid in, for instance, evaluating and developing theories and, therewith, developing evidence-based treatments and policy.

## 1. Introduction

To answer important societal questions, aggregation of results from multiple independent studies investigating the same theoretical relationship is needed. If multiple studies investigate the same theoretical relationship, the results of these studies together offer more certainty about that relationship in the population than the result of any single study. One often used method to systematically summarize quantitative results from multiple studies is meta-analysis, a secondary data-analysis on estimates of primary studies. Meta-analysis aims to aggregate effect-size estimates—like standardized mean difference(s), correlation(s), odds ratio(s), or standardized regression parameters—from multiple studies to come to an overall estimate of one or more population parameters with confidence intervals and standard errors [1,2].

Typically, researchers use meta-analysis models to assess the population relationships between pairs of variables through the estimation of parameters. Currently, meta-analyses may lack theory development, but by offering a method that can evaluate a priori expectations directly, as done in this paper, meta-analytic researchers may shift their focus to theory development. Therefore, I assume—in the remainder of this paper—that the primary aim of a meta-analysis is to compare the size and/or sign of the effect size parameters For example, one may want to examine the relationship between the outcome buyer–seller trust and the three predictors worth of previous transactions, the worth of expected future transactions, and the size of the buyer’s network; which can be reflected by the population (standardized) parameters $\theta_{1}$, $\theta_{2}$, and $\theta_{3}$, respectively. Then, one may expect beforehand an ordering in the importance of predictors and possibly (also) of moderators; for instance, one may hypothesize that $\theta_{1}<\theta_{2}<\theta_{3}$.

Many theories can be expressed by hypotheses with inequality constraints on the population parameters (e.g., $\theta_{1}<\theta_{2}<\theta_{3}$ or even $|\theta_{1}\left|\;<\;\right|\theta_{2}\left|\;<\;\right|\theta_{3}$|, where |.| denotes the absolute value), as illustrated in Appendix A.1.1, Appendix A.1.2, Appendix A.1.3. Nevertheless, statistical hypotheses are often expressed in terms of hypotheses containing solely equality constraints (e.g., $\theta_{1}=\theta_{2}=\theta_{3}$), because these hypotheses can be tested with null hypothesis tests or evaluated with model selection using, for instance, the Akaike’s information criterion (AIC).

Even in combination with the sizes of the estimates, null hypothesis testing does not properly address the theory-based hypotheses like $\theta_{1}<\theta_{2}<\theta_{3}$; nor does model selection using the AIC. What if ‘$\theta_{1}=\theta_{2}=\theta_{3}$’ is rejected? Or what if ‘$\theta_{1}=\theta_{2}$’ is rejected and ‘$\theta_{2}=\theta_{3}$’ is not? What if ‘$\theta_{1},\;\theta_{2},\;\theta_{3}$’ is the best from the set of candidate models? What should be concluded? Can the result(s) then be expressed in terms of support for or against the hypothesis of interest? Often it cannot. Hence, one would want to evaluate the hypothesis of interest ($\theta_{1}<\theta_{2}<\theta_{3}$) directly. Fortunately, it is possible to evaluate theory-based hypotheses by using the AIC-type inequality-constrained model selection criterion called generalized order-restricted information criterion approximation (GORICA) [3,4,5].

This paper will show how the GORICA can evaluate theory-based hypotheses (which probably include inequality constraints) in a meta-analytic study. The GORICA will then be applied to meta-analyzed estimates. These estimates can be effect size measures like Cohen’s *d* or Hedges’ *g* but can also be standardized estimates from, for example, (multivariate) regression models or logistic regression models and/or they can be the standardized estimates of moderators (see Appendix A.1.1, Appendix A.1.2, Appendix A.1.3 for more details).

Next, I will give some background information on meta-analysis and on how one can test or evaluate hypotheses using null hypothesis tests, the AIC, and the GORICA. This is followed by a section regarding meta-analytic examples, where I will show input and output for applying null hypothesis tests, the AIC, and the GORICA to meta-analyzed estimates in R [6]. In addition, I will compare the usability of the output of the GORICA (evaluating theory-based hypotheses) versus that of null hypothesis tests and the AIC (evaluating null hypotheses). I will conclude with a discussion regarding the (dis)advantages of the GORICA compared to some other methods.

## 2. Materials & Methods: Meta-Analysis

In this section, the core concepts of meta-analysis are briefly introduced. The remainder of this paper focuses on illustrating how theory-based hypotheses, often containing inequality constraints, can be evaluated using null hypothesis testing, the AIC, and the GORICA.

Meta-analysis aims to aggregate evidence from several different studies, usually in the form of a statistical parameter or set of parameters (e.g., an effect size measure or standardized regression parameters) estimated from different samples, to come to an overall estimate of one or more population parameters [1,2]. In principle, meta-analysis combines evidence from different studies by taking a weighted average of the parameter estimates, where the study weights reflect the amount of information or certainty in a given estimate. This weighting procedure can be applied *univariately* (via weighted least squares; using the inverse of the variance of one or more estimates); or *multivariately* (via generalized least squares; using the inverse of the covariance matrix of the estimates) to take into account dependencies between the parameters which are the target of the meta-analysis. The latter is comparable to the method called parameter-based meta-analytic structural equation modeling (MASEM) (cf. [7]). For a more in-depth treatment of these weighting procedures, the reader is referred to Becker & Wu [2] and Demidenko and colleagues [8].

Another distinction in meta-analytic techniques can be made based on the assumed underlying population parameter model: Researchers can assume a single underlying parameter, in a *fixed-effect* analysis (also referred to as common-effect or equal-effect model), or a distribution of population parameters, in a *random-effects* model. The latter reduces to the former if all variance in each of the parameter estimates is assumed to come from sampling variance alone. Note that a random-effects model should be used to generalize meta-analytic results beyond the primary studies included in the meta-analysis. For an elaboration on meta-analytic techniques, see for instance Borenstein and colleagues [1] and Becker & Wu [2].

Additionally, one can include moderators, that is, predictors on the study-level, in a (multiple) *meta-regression*. Moderators can be used to explain some of the heterogeneity variances in the meta-analysis (which may be due to differences in study designs or, as another example, publication-year). Note that subgroup analysis is a special case, since then the predictors are dummy/grouping/categorical variables. In this model, it is assumed that not all studies stem from the same population (because of different study characteristics) and that the true overall effects differ per subgroup (or vary with the moderator values). Such a model is also referred to as moderator analysis or (multilevel) fixed-effects plural model or (multilevel) *mixed-effects* model.

All these types of meta-analysis models are captured by the following equation:

$${\hat{\theta }}_{s}=\theta +\beta \;x_{s}+\epsilon_{s}+\zeta_{s},$$

where ${\hat{\theta }}_{s}$ is the observed effect size(s) of Study *s* (which can consist of multiple elements), $x_{s}$ is the moderator(s), $\epsilon_{s}$ is the sampling error (how much does the effect size deviate from its true effect), and $\zeta_{s}$ is the random effect denoting between-study heterogeneity (implying that the true effect size comes from an overarching distribution of effect sizes), which is independent from the sampling error. In case there are no moderators, ‘$\beta \;x_{s}$’ is left out in the equation or, stated otherwise, β is assumed to be 0. In case there is no random effect (i.e., there is solely a fixed effect), ‘$\zeta_{s}$’ is left out in the equation; stated otherwise, the variance of $\zeta_{s}$ (often referred to as $\tau^{2}$) is assumed to be zero.

In principle, meta-analysis takes a *weighted* average of the effect-size estimates, where the contribution of each study is weighted by the inverse of the variance of the estimate (or the inverse of the covariance matrix of the estimates), that is, the amount of information or certainty in the estimate(s). To be more precise, the study contribution is weighted by the inverse of the sampling variance (matrix) in the case of a fixed-effect model and by the inverse of the total variance (matrix) in the case of a random-effects model.

### Software

There are several software programs to perform a meta-analysis. In this manuscript, I will make use of the R package metafor [9].

Next, I will describe some currently used methods to test (null) hypotheses, the differences between these tested (null) hypotheses and the ones of interest, and how the GORICA can help out.

## 3. Materials & Methods: Null Hypothesis Evaluation

In Appendix A, I describe and exemplify different types of hypotheses that may be of interest in meta-analysis (based on four meta-analysis cases) and the ones that can be addressed in null hypothesis evaluation methods. As an example (which was also used in the introduction), one may be interested in $\theta_{1}<\theta_{2}<\theta_{3}$, while null hypothesis evaluation methods will evaluate $\theta_{1}=\theta_{2}=\theta_{3}$ (and, possible, some or all pairwise combinations of equality restrictions). Next, I describe two methods that evaluate null hypotheses: Null hypothesis testing and the model selection criterion AIC. Subsequently, I describe some of their downsides.

### 3.1. Null Hypothesis Tests

Null hypotheses, like $\theta_{1}=\theta_{2}=\theta_{3}$, can be tested with null hypotheses tests: by using a *t* test, an *F* test, or a (Wald-type) $\chi^{2}$ test—all leading to a *p*-value—and/or by inspecting the (elliptical, that is, multivariate) confidence intervals. Unfortunately, a *p*-value and (the width of) confidence intervals do not quantify the support for any hypothesis. Note that under a severe testing view of the evidence, a *p*-value is interpreted in terms of the probability of obtaining evidence as strong as the observed data provides purely by chance (cf. [10]). Luckily, model selection can be used to not only evaluate restrictions on parameters simultaneously but also to quantify the support of hypotheses, as discussed in Section 3.2.

#### Software

The R package metafor renders output for null hypothesis testing. This will be demonstrated in the Illustrations section (i.e., Section 5).

### 3.2. Model Selection Using the AIC

The evaluation of a hypothesis against one or more competing ones can be done with model selection. One type of model selection is information-theoretical model selection, which uses information criteria. Model selection techniques, like information criteria, select the hypothesis that describes the data best (highest fit) with the smallest (least complex) hypothesis in terms of the number of distinct parameters, out of a set of candidate hypotheses.

An often-used information criterion is the Akaike Information Criterion (AIC) [11]. The AIC is an estimate of the Kullback–Leibler discrepancy [12], the distance between a candidate hypothesis and the true unknown hypothesis. Therefore, the hypothesis with the smallest AIC value is the preferred one in the set of candidate hypotheses. The AIC quantifies the trade-off between the fit (likelihood) and the complexity (penalty) of the candidate hypotheses in the following way:

AIC=−2{maximum log likelihood−penalty},

where the penalty equals the number of distinct model parameters: e.g., the number of distinct regression parameters, including the intercept, and the distinct error (co)variance(s).

The AIC can evaluate null hypotheses, like $\theta_{1}=\theta_{2}=\theta_{3}$ and $\theta_{1}=\theta_{2},\;\theta_{3}$. Moreover, it can compare multiple hypotheses (containing equality restrictions). For instance, one can compare the following three hypotheses:

$$\begin{array}{ccc} H_{01}: & & \theta_{1}=\theta_{2}=\theta_{3},\\ H_{02}: & & \theta_{1},\;\theta_{2}=\theta_{3},\\ H_{u}: & & \theta_{1},\;\theta_{2},\;\theta_{3}, \end{array}$$

where $H_{01}$ restricts all parameters to be the same; $H_{02}$ restricts only the last two parameters to be equal and does not restrict $\theta_{1}$, that is, it freely estimates $\theta_{1}$; and $H_{u}$ freely estimates all three parameters. The hypothesis with the smallest AIC is then the preferred one.

#### Software

While meta-analysis results can be accompanied with an AIC value, most meta-analytic software cannot constrain estimates (within a model). Note that the AIC in the R package metafor is only helpful (1) when comparing different random effects structures; (2) when comparing a random-effects meta-analytic model with a fixed-effect model; or (3) when comparing models with different sets of fixed effects (in case all studies have observed data for all the fixed effects, otherwise the data sets differ per model). In these cases, one has to specify and run the models/hypotheses of interest separately and ask for the AIC values for those models. To learn more about how well information criteria (like the AIC) perform in model selection in meta-analysis, the interested reader is referred to [13].

Since meta-analytic software cannot restrict meta-analyzed estimates, not all types of models/hypotheses can be compared with the rendered AIC. One can, for instance, not evaluate whether the parameters for multiple outcomes are zero or any other value (as evaluated in the Illustrations section, that is, Section 5). To evaluate such equality-restricted restrictions, the so-called GORICA weights (as discussed in the next section) will be calculated as a proxy to the Akaike weights and referred to as AIC weights.

### 3.3. Gap and Bridging the Gap

Equality-restricted hypotheses evaluated in null hypothesis evaluation often differ from the theory-based hypotheses (which often contain inequality restrictions), as can be seen when comparing Appendix A.1 to Appendix A.2. Even in combination with the sizes of the estimates, null hypothesis testing does not properly address the theory-based hypotheses; nor does model selection (on equality-constrained hypotheses) using the AIC. Fortunately, it is possible to evaluate theory-based hypotheses by using the AIC-type inequality-constrained model selection criterion called GORICA.

Next, I give some background information on the GORICA. This is followed by a section regarding meta-analytic examples. There, I will show R input and output for doing null hypothesis tests, the AIC, and the GORICA and compare their usability.

## 4. Materials & Methods: GORICA

By using the generalized order-restricted information Criterion (GORIC) [4,5] or its approximation (GORICA) [3], researchers’ theories can directly be examined by evaluating theory-based hypotheses, like $\theta_{1}=\theta_{2}>\theta_{3}$ or $\theta_{1}>\theta_{2}>\theta_{3}$. Thus, the GORIC and GORICA can evaluate theory-based hypotheses containing order restrictions on the parameters (“<” and/or “>”) besides equality restrictions (“=”). They can evaluate hypotheses with restrictions on linear combinations of parameters (notably, a restriction regarding the square of say $\theta_{1}$ is not possible, but this is oftentimes also not of interest).

The GORIC is an extension of the AIC (and, thus, also an estimate of the Kullback–Leibler discrepancy) and is of the form

GORIC=−2{maximum order-restricted log likelihood−penalty}.

This expression is based on the order-restricted maximum likelihood (i.e., the maximum likelihood under the order restrictions in the hypothesis) and has a more general penalty expression (using so-called chi-bar-square weights) such that the order restrictions are properly accounted for (for more details, see Appendix C). The penalty equates, loosely speaking, the expected number of distinct parameters. For example, $\theta_{1}<\theta_{2}$ represents 1.5 distinct regression parameters and not 2, as would be the case in the AIC (which would evaluate $\theta_{1},\;\theta_{2}$). If there are solely equality constraints (“=”) and/or no constraints (“,”) and, thus, no order restrictions, the GORIC reduces to the AIC.

The GORICA is an approximation method which eases the calculation of the GORIC for a broad range of models. It uses the fact that maximum likelihood estimates are asymptotically normally distributed:

$$GORICA=-2\;\{LL_{MLE}-penalty\},$$

where $LL_{MLE}$ is the (order-restricted) fit part (different from that of the GORIC) and where ‘penalty’ is the (order-restricted) complexity part which has the same expression as that of the GORIC; where both parts account for the order restrictions in the hypothesis. The fit part of the GORICA, $LL_{MLE}$, is (besides the order restrictions) based on the maximum likelihood estimates (MLEs) and their covariance matrix (which are a summary of the data) instead of the data themselves. Using the central limit theory, the fit part of the GORICA is always based on the normal distribution for the MLEs even if the data do not follow one (like in a logistic regression). The fit part is the maximum of this distribution given the restrictions in the theory-based hypotheses (i.e., it is an order-restricted maximum). The interested reader is referred to Appendix C for details about the log likelihood and penalty parts of the GORIC and GORICA.

Because of the different fit expressions, the fit values of the GORIC and GORICA differ in an absolute sense but asymptotically not in the relative sense when comparing candidate hypotheses (cf. [3] and Appendix C). Therefore, the GORICA asymptotically selects the hypothesis with the smallest distance to the truth (while the GORICA value itself is not an estimate of the Kullback–Leibler discrepancy). The GORICA, like the AIC and GORIC, orders the hypothesis in the set, where the hypothesis with the smallest value is the preferred one.

Note that the GORICA only needs the estimates of the (unconstrained) parameters of interest and their covariance matrix. To be more precise, the estimates and their covariance matrix are only needed for the parameters included in the set of hypotheses, which often do not include variance components (for more details, see Appendix C). Therefore, it can easily be applied to all types of meta-analyzed estimates (like effect-size measure estimates or standardized regression estimates), as long as their covariance matrix is also known (which is often, if not always, part of meta-analytic software).

### 4.1. GORICA Weights

To improve the interpretation of information criteria values, one should transform them into weights. The GORICA weight for Hypothesis $H_{m}$ is calculated by:

$$w_{m}=\frac{exp\left(-\frac{1}{2}\;GORICA_{m}\right)}{\sum_{m^{\prime }=1}^{M}exp\left(-\frac{1}{2}\;GORICA_{m^{\prime }}\right)}$$

for i=1,…,M, with *M* the total number of hypotheses in the set and $\sum_{m=1}^{M}w_{m}=1$. Notably, the GORICA weights and the GORIC weights are asymptotically the same. In the case of no order restrictions, the GORIC weights equal the Akaike weights (cf. [14]) and, thus, the GORICA weights asymptotically equal Akaike weights (and can thus be used a proxy).

Bear in mind that an information criterion (IC) can be written as

IC=−2{LL−penalty}.

Consequently,

$$exp\left\{-\frac{1}{2}\;IC\right\}=exp\left\{LL-penalty\right\}=exp\left\{LL\right\}/exp\left\{penalty\right\}=likelihood/exp\left\{penalty\right\}.$$

Hence, the IC weights are comparable to likelihood ratios, only now the complexity of the hypotheses/models are also taken into account.

The IC weights reflect the strength/likelihood/support of a hypothesis given the data and the set of hypotheses [4,14,15,16]. That is, $w_{m}$ denotes the weight of evidence that Hypothesis $H_{m}$/Model *m* is the best hypothesis for the data at hand given the *M* candidate hypotheses. Thus, when inspecting another set of hypotheses, the weights for the same hypotheses may change.

For the comparison of two hypotheses, one can use the ratio of their weights, denoting the relative support of one hypothesis versus the other. For instance, GORICA weights for Hypothesis $H_{m}$ and a competing hypothesis $H_{c}$ of $w_{m}=0.875$ and $w_{c}=0.125$ mean that $H_{m}$ has $w_{m}/w_{c}=0.875/0.125=7$ times more support than the competing hypothesis $H_{c}$. Stated otherwise, $H_{m}$ is 7 times more likely than the competing hypothesis $H_{c}$. For readers who are familiar with Bayesian statistics, the information criterion weights (e.g., GORICA weights) are comparable to posterior model probabilities and the relative support (i.e., ratio of weights) to Bayes factors. Note that the relative support (i.e., ratio of weights) does not depend on the full set of candidate models, it is the support for one hypothesis relative to one other hypothesis. For example, when researchers would include an additional hypothesis in the set, they may find weights of 0.7, 0.1, and 0.2, but the relative support of $H_{m}$ vs. $H_{c}$ still equals 7, namely 0.7/0.1 = 7.

### 4.2. Software

There are two R functions that can calculate GORICA values and weights: the goric function [17] in the restriktor package [18] and the gorica function in the gorica package [19]. These functions render the same results of course, but there are some differences in functionality (cf. [20]). The goric function of the restriktor package is used in this paper.

The next section demonstrates, among other things, how the GORICA can be applied to meta-analyzed parameter estimates and gives insight into the (dis)advantages of using the GORICA. It also contains remarks for specific types of hypotheses, which are explicitly and more elaborately addressed in Appendix B.

## 5. Results: Illustrations

I will make use of empirical meta-analytic studies, by using datasets provided by the site of Wolfgang Viechtbauer (accessed on 1 June 2022). For several data sets, I run a meta-analysis including null hypothesis testing in R [6] and I applied both the AIC and GORICA to the meta-analyzed estimates. An R script containing annotated R code is available on my GitHub page (accessed on 1 June 2022). These include meta-analyses regarding effect size measures and model parameters, both with and without moderators (i.e., examples for each of the four cases discussed in Appendix A). For brevity, I will next show (a part of) one of the meta-analyses (including R code). Based on that, I will give insight in the comparison of evaluating null hypothesis in meta-analysis (using null hypothesis testing and the AIC) with the proposed theory-based hypothesis evaluation using the GORICA.

In this section, the meta-analytic study of Berkey and colleagues [21] is used, where surgical and non-surgical treatments for medium-severity periodontal disease is compared in five trials for two outcomes: attachment level (AL) and probing depth (PD) one year after the treatment. In this meta-analysis, the effect size to be aggregated is the (raw) mean difference, where non-surgical treatment is the reference category. This means that a positive value indicates that surgery was more effective than non-surgical treatment. Note that the outcomes are negatively related: a positive estimate indicates effectiveness of surgery in either increasing the attachment level or decreasing the probing depth.

Meta-analysis is used to obtain an estimate of the population mean differences, where the latter will be denoted by the population parameters $\theta_{AL}$ and $\theta_{PD}$. From theory, it might be expected that the first parameter is negative and the latter positive, leading to the following hypothesis of interest:

$$\begin{array}{ccc} H_{1.1}: & & \theta_{AL}<0,\;\theta_{PD}>0. \end{array}$$

In the case that there might also be reason to believe that $\theta_{PD}$ could be negative, there is a competing hypothesis of interest:

$$\begin{array}{ccc} H_{1.2}: & & \theta_{AL}<0,\;\theta_{PD}<0. \end{array}$$

Alternatively, it can be the case that there is theory regarding the size of the effect size, or one wants to compare it to a cut-off value for that specific effect size type (e.g., when using Cohen’s d or Hedges’ *g*). In this example, there might be theory regarding the size of the mean difference stating:

$$\begin{array}{ccc} H_{2}: & & |\theta_{AL}\left|\;>\;0.2,\;\right|\theta_{PD}|\;>\;0.2, \end{array}$$

where |x| denotes the absolute value of *x*.

As another example, it might be expected from theory that the absolute size of $\theta_{AL}$ is smaller than that of $\theta_{PD}$:

$$\begin{array}{ccc} H_{3}: & & |\theta_{AL}\left|\;<\;\right|\theta_{PD}|. \end{array}$$

Note that, for a fair comparison of parameters, both outcomes should be on the same scale (as is the case here). Comparing sizes can be meaningful in the case of multiple outcomes, like here, or in the case of multiple (aggregated) standardized regression estimates (where one then compares the importance of the corresponding predictors).

Next, the input and (part of the) output for the meta-analysis is given. Subsequently, one can find the results and conclusions regarding hypotheses $H_{1.1}$ to $H_{3}$ when performing null hypothesis testing, model selection using the AIC, and model selection using the GORICA.

### 5.1. Meta-Analysis

The R code to (multivariately) aggregate the estimates of the five trials with a meta-analysis using the metafor package:

# data

data <-~dat.berkey1998

# Covariance matrix, needed for multivariate meta-analysis

V <- bldiag(lapply(split(data[,c("v1i", "v2i")], data$trial), as.matrix))

# meta-analysis

metaan <- rma.mv(yi, V, mods = ~ outcome - 1, random = ~ outcome | trial,

struct="UN", data=data, method="ML")

print(metaan, digits=3)

Note that the function rma.mv() uses restricted maximum likelihood (REML) estimation by default. Hence, method = “ML” must be explicitly requested, which is used to mimic [21].

This renders output. The, for this paper, relevant part of the output is:

Model Results:

estimate    se    zval   pval   ci.lb   ci.ub

outcomeAL     -0.338   0.080    -4.237   <.001   -0.494   -0.182  ***

outcomePD     0.345   0.049    6.972   <.001   0.248   0.442  ***

---

Signif. codes:  0 ‘***’ 0.001 ‘**’ 0.01 ‘*’ 0.05 ‘.’ 0.1 ‘ ’ 1

Be aware that the theory-based hypotheses are (and should be) formulated before inspecting this.

### 5.2. Null Hypothesis Testing

In the example where $H_{1.1}:\theta_{AL}<0,\;\theta_{PD}>0$ (and possible $H_{1.2}:\theta_{AL}<0,\;\theta_{PD}<0$) is of interest, there are two null hypotheses, namely surgery was equally effective as non-surgical treatment in increasing the attachment level and in decreasing the probing depth. This can be represented by the following two statistical hypotheses:

$$\begin{array}{ccc} H_{0a}: & & \theta_{AL}=0\\ H_{0b}: & & \theta_{PD}=0. \end{array}$$

Classical null hypothesis tests are part of the meta-analysis output, as can be seen in the previous subsection. From this, it can be concluded that both hypotheses can be rejected (*p* < 0.001). Thus, there is a significant difference in the effectiveness of the treatments for both outcomes. When inspecting the sign of the meta-analyzed estimates, it can be concluded that, on average, surgery is more effective in decreasing probing depth (i.e., 0.35 > 0) and less effective in increasing the attachment level (i.e., −0.34 < 0) than non-surgery (i.e., non-surgery is more effective in increasing the attachment level). Note that one can also do a one-tailed test to test, for instance, $\theta_{AL}>0$ (by dividing the *p*-value by two, when the sign is in agreement with the expectation; otherwise, one should use 1−p/2).

In the above, the two null hypotheses are not tested simultaneously. One may like to test them simultaneously by testing the following null hypothesis:

$$\begin{array}{ccc} H_{0}: & & \theta_{AL}=0,\;\theta_{PD}=0. \end{array}$$

This null can be tested by inspecting elliptical/multivariate confidence intervals (which are based on the covariance matrix of the meta-analyzed estimates), but only the univariate confidence intervals are reported when using metafor. Alternatively, one can use a chi-square test. When using metafor, one can test $H_{0}$ with the (Wald-type) chi-square test:

# R code

anova(metaan)

# Output:

Test of Moderators (coefficients 1:2):

QM(df = 2) = 155.7728, p-val < 0.0001

This omnibus test renders a *p*-value smaller than 0.001, indicating that $H_{0}$ is rejected. If of interest, one can test a null hypothesis for a specific set of parameters via the btt argument in the anova function (e.g., ‘anova(metaan, btt = 1:2)’ for the first two parameters). Note that one cannot test $H_{1.1}:\theta_{AL}<0,\;\theta_{PD}>0$, since there is no such one-tailed test.

Thus, even when testing the null hypotheses simultaneously (by testing $H_{0}$ directly), this cannot address the hypotheses of interest (especially when there are more than two parameters). In this analysis with two parameters, it does give some insight into $H_{1.1}$ and perhaps $H_{1.2}:\theta_{AL}<0,\;\theta_{PD}<0$, when also inspecting the meta-analyzed estimates. Namely, $H_{1.1}$ seems to be supported by the results, while $H_{1.2}$ is not or only partly. However, it is not clear how large their support is. Additionally, one cannot compare (the support for) these two hypotheses.

When inspecting $H_{2}:|\theta_{AL}\left|\;>\;0.2,\;\right|\theta_{PD}|\;>\;0.2$ and $H_{3}:|\theta_{AL}\left|\;<\;\right|\theta_{PD}|$, one can test ‘$\theta_{AL}=0.2,\;\theta_{PD}=0.2$’ and ‘$\theta_{AL}=\theta_{PD}$’, respectively. One again has to additionally inspect the meta-analyzed estimates to obtain more insight into the hypotheses of interest. Additionally, one still cannot quantity the support for the hypotheses of interest: In general, a *p*-value and (the width of) elliptical confidence intervals do not quantify the support for any hypothesis.

As will be demonstrated next, model selection can be used to evaluate restrictions on parameters simultaneously and quantify the (relative) support of the hypotheses included in the set.

### 5.3. Null Hypothesis Selection (Using the AIC)

To evaluate and compare null hypotheses, one can conduct model selection using the AIC value. Because metafor cannot render the AIC values for the null hypotheses of interest in this example, I will use the GORICA weights as a proxy to the Akaike weights and refer to them as AIC weights. Note that this way I can still compare model selection evaluation of null hypotheses versus that of theory-based hypotheses.

To apply the GORICA to meta-analyzed estimates in R, these estimates and their covariance matrix should be extracted:

#Substract estimates from meta-an, to~be used in goric function

est <- coef(metaan)

names(est) <- c("theta_AL", "theta_PD")

VCOV_est <- vcov(metaan)

In this analysis with two parameters, the number of possible equality hypotheses is not that large. Therefore, all possibilities will be inspected here. To prevent choosing the best from a set of weak/bad hypotheses (i.e., from a set of hypotheses not supported by the data), the unconstrained hypothesis (which does not restrict parameters) is included in the set as a fail-safe. Stated otherwise, when the equality-restricted hypotheses are not supported by the data, the unconstrained hypothesis will be the best of the set. See Appendix B.2 for more information.

Notably, in the case of more than two parameters, one may want to reduce the number of hypotheses, like Burnham & Anderson [14] also recommend. This should then be based on theory. As a side note, in case there is theory, I expect a researcher to have expectations which will most probably include order restrictions as is the case in hypotheses $H_{1.1}$ to $H_{3}$ above (e.g., $H_{1.1}:\theta_{AL}<0,\;\theta_{PD}>0$). In such a case, one should evaluate these directly with GORICA, as will become clear later.

Based on the three example sets of hypotheses specified, the following three hypothesis sets are used. The first set compares each of the two parameters to zero:

$$\begin{array}{ccc} H_{01}: & & \theta_{AL}=0,\;\theta_{PD}=0\\ H_{02}: & & \theta_{AL},\;\theta_{PD}=0\\ H_{03}: & & \theta_{AL}=0,\;\theta_{PD}\\ H_{unc}: & & \theta_{AL},\;\theta_{PD}. \end{array}$$

The second set compares each of the two parameters to 0.2 (in absolute sense):

$$\begin{array}{ccc} H_{04}: & & |\theta_{AL}\left|=0.2,\;\right|\theta_{PD}|=0.2\\ H_{unc}: & & \theta_{AL},\;\theta_{PD}. \end{array}$$

Note that, when using the GORICA, one can also evaluate restrictions regarding absolute values of parameters. The third set compares the parameters to each other (in absolute sense):

$$\begin{array}{ccc} H_{0}: & & |\theta_{AL}\left|=\right|\theta_{PD}|\\ H_{unc}: & & \theta_{AL},\;\theta_{PD}. \end{array}$$

In R, using the goric function in the restriktor package, these three hypotheses sets are formulated as follows:

# Set 1

H01 <- "theta_AL == 0; theta_PD == 0"

H02 <- "theta_PD == 0"                # i.e.,~theta_AL, theta_PD == 0

H03 <- "theta_AL == 0"                # i.e.,~theta_AL == 0, theta_PD

# Note: By default, the~unconstrained hypothesis is added to the~set.

# Set 2

H04 <- "abs(theta_AL) == 0.2; abs(theta_PD) == 0.2"

# Note: This can be compared to its complement, but~because of the equality,

# the complement equals the unconstrained~hypothesis.

# Set 3

H05 <- "abs(theta_AL) == abs(theta_PD)"

# Note: This can be compared to its complement, but~because of the equality,

# the complement equals the unconstrained~hypothesis.

The following R code should be used to evaluate these sets with the GORICA:

# Apply GORICA to obtain AIC~weights

# Set 1

results_AIC_Set1 <- goric(est, VCOV = VCOV_est, H01, H02, H03,

type = "gorica")

results_AIC_Set1

# Set 2

results_AIC_Set2 <- goric(est, VCOV = VCOV_est, H04,

comparison = "complement", type = "gorica")

results_AIC_Set2

# Set 3

results_AIC_Set3 <- goric(est, VCOV = VCOV_est, H05,

comparison = "complement", type = "gorica")

results_AIC_Set3

The output next shows the AIC weights (‘gorica.weights’) for the first set of hypotheses without order restrictions. Note that the reported log likelihood (loglik) and penalty are based on the structural parameters, that is, the parameters included in the set of hypotheses. For more details, see Appendix C.

Results:

model   loglik  penalty   gorica  gorica.weights

1            H01  -73.975    0.000  147.950           0.000

2            H02  -20.393    1.000   42.787           0.000

3            H03   -5.063    1.000   12.127           0.000

4  unconstrained         3.912    2.000   -3.824           1.000

---

From this, it is concluded that the unconstrained hypothesis is the best hypothesis, since it has the smallest IC value and the largest IC weight. It even has full support, reflected by an IC weight of 1. This implies that the other three hypotheses are weak hypotheses, that is, hypotheses not supported by the data. Note that these three hypotheses (i.e., $H_{01}$ to $H_{03}$) do not reflect any of the mentioned possible hypotheses of interest (i.e., $H_{1.1}:\theta_{AL}<0,\;\theta_{PD}>0$ and $H_{3}:|\theta_{AL}\left|<\right|\theta_{PD}|$) which are included in the unconstrained. Based on the results, one can conclude that there is overwhelming support that both estimates are not zero. When inspecting the signs of the meta-analyzed estimates, something can be said about $H_{1.1}:\theta_{AL}<0,\;\theta_{PD}>0$ and $H_{1.2}:\theta_{AL}<0,\;\theta_{PD}<0$, but one cannot quantify the support for these hypotheses or the support relative to each other.

The output next shows the AIC weights (‘gorica.weights’) for the second set of hypotheses without order restrictions:

Results:

model  loglik  penalty  gorica  gorica.weights

1         H04  -9.560    0.000  19.120           0.000

2  complement       3.912    2.000  -3.824           1.000

---

The order-restricted hypothesis ‘H04’ has  0.000 times more support

than its complement.

From this, it is concluded that there is no support for $H_{04}:|\theta_{AL}\left|=0.2,\;\right|\theta_{PD}|=0.2$. Thus, there is full support for the unconstrained, the complement of $H_{04}$. However, the unconstrained contains both values above and below 0.2. Thus, one should perhaps inspect the size of the meta-analyzed estimates. Although this renders more insight, it still lacks support for the hypothesis of interest $H_{2}:|\theta_{AL}\left|>0.2,\;\right|\theta_{PD}|>0.2$.

The output next shows the AIC weights (‘gorica.weights’) for the third set of hypotheses without order restrictions:

Results:

model  loglik  penalty  gorica  gorica.weights

1         H05   3.910    1.000  -5.820           0.731

2  complement      3.912    2.000  -3.824           0.269

---

The order-restricted hypothesis ‘H05’ has  2.713 times more support

than its complement.

From this, it is concluded that $H_{05}:|\theta_{AL}\left|=\right|\theta_{PD}|$ has 0.731/0.269≈2.7 times more support than the unconstrained hypothesis (which includes $H_{05}$). This may not seem to be convincing evidence, but it is. This has to do with evaluating an equality which is almost true in the data (judged by the two almost equal log likelihood (loglik) values), as discussed in Appendix B.4. As was the case in null hypothesis testing, inspecting the meta-analyzed estimates renders more insight, but it still lacks support for the hypothesis of interest $H_{3}:|\theta_{AL}\left|<\right|\theta_{PD}|$.

The examples above show that the AIC can quantify the support of the hypotheses in the set, where multiple parameters can be constrained simultaneously. Nevertheless, the hypotheses in the set are not per se the hypotheses a researcher is interested in. In such a case, a researcher can still not quantity the support for the hypotheses of interest and/or compare their support. As will be shown next, this is possible when evaluating order-restricted hypotheses with the GORICA.

### 5.4. GORICA

The GORICA can evaluate hypotheses with (and without) order restrictions. Hence, it can directly evaluate Hypotheses $H_{1.1}$ to $H_{3}$ mentioned above (e.g., $H_{1.1}:\theta_{AL}<0,\;\theta_{PD}>0$). To apply the GORICA to meta-analyzed estimates in R, these estimates and their covariance matrix should be extracted:

#Substract estimates from meta-an, to~be used in goric function

est <- coef(metaan)

names(est) <- c("theta_AL", "theta_PD")

VCOV_est <- vcov(metaan)

Next, different sets of theory-based hypotheses are evaluated.

Hypothesis $H_{1.1}:\theta_{AL}<0,\;\theta_{PD}>0$ can be evaluated against its complement (see Appendix B.2 for more information) with the following R code:

# Hypothesis of interest

H1.1 <- "theta_AL < 0; theta_PD > 0"

# Apply GORICA

set.seed(123) # set seed: to obtain the same results when you re-run it

results_H1.1 <- goric(est, VCOV = VCOV_est, H1.1,

comparison = "complement", type = "gorica")

results_H1.1

The corresponding output is:

Results:

model  loglik  penalty  gorica  gorica.weights

1        H1.1   3.912    0.799  -6.226           1.000

2  complement      -5.063    1.701  13.529           0.000

---

The order-restricted hypothesis ‘H1.1’ has 19482.703 times more support

than its complement.

From this, it can be concluded that the hypothesis of interest $H_{1.1}$ has full support (when compared to any other ordering/theory). Thus, there is overwhelming support for the hypothesis of interest stating that surgery is less effective in increasing the attachment level than non-surgery and more effective in decreases in probing depth.

Now, assume a researcher is not only interested in $H_{1.1}:\theta_{AL}<0,\;\theta_{PD}>0$ but also in the competing hypothesis $H_{1.2}:\theta_{AL}<0,\;\theta_{PD}<0$. Since the two hypotheses do not cover the whole space/do not cover all possible orderings of parameters, one should include the unconstrained hypothesis to prevent choosing a weak/bad hypothesis (see Appendix B.2 for more information). This can be done using the following code:

# Hypothesis of interest

H1.1 <- "theta_AL < 0; theta_PD > 0"

H1.2 <- "theta_AL < 0; theta_PD < 0"

# Note: By default, the~unconstrained hypothesis is added to the~set.

# Apply GORICA

set.seed(123) # set seed: to obtain the same results when you re-run it

results_H1 <- goric(est, VCOV = VCOV_est, H1.1, H1.2,

type = "gorica")

results_H1

round(results_H1$ratio.gw, digits = 2)

This results in the following output:

Results:

model   loglik  penalty  gorica  gorica.weights

1           H1.1    3.912    0.799  -6.226           0.769

2           H1.2  -20.393    1.201  43.189           0.000

3  unconstrained    3.912    2.000  -3.824~0.231

> round(results_H1$ratio.gw, digits = 2)

vs. H1.1       vs. H1.2   vs. unconstrained

H1.1               1.0    53728634328                3.32

H1.2               0.0              1                0.00

unconstrained      0.3    16165185252                1.00

From this, one can conclude that $H_{1.1}$ is not a weak hypothesis since it is (3.3 > 1 times) more supported than the unconstrained hypothesis; and that $H_{1.2}$ is weak (since it is 0 or, to be more precise, $6\times 10^{-11}<<1$ times more supported than the unconstrained), that is, $H_{1.2}$ is not supported by the data. Because at least one of the hypotheses of interest is not weak, these hypotheses can be meaningfully compared to each other, which is the interest in this example. It can be concluded that $H_{1.1}$ is many more (nl. 53,728,634,328) times supported than $H_{1.2}$.

Note that the unconstrained hypothesis includes all possible hypotheses and, thus, also $H_{1.1}:\theta_{AL}<0,\;\theta_{PD}>0$. Therefore, the support for the unconstrained includes support for $H_{1.1}$. If one would leave out the unconstrained (which is only included as a safeguard), $H_{1.1}$ would have full support (i.e., an IC weight of 1) here, which is also reflected by the relative GORICA weights (i.e., 53,728,634,328). Thus, the results for the set excluding the unconstrained can be inferred from the one including the unconstrained (for this, one could also use the R function IC.weights [22] when necessary).

In conclusion, there is overwhelming support for the hypothesis of interest stating that surgery is less effective in increasing the attachment level than non-surgery and more effective in decreases in probing depth, compared to stating that surgery is less effective in increasing the attachment level than non-surgery and less effective in decreases in probing depth.

In case $H_{2}:|\theta_{AL}\left|>0.2,\;\right|\theta_{PD}|>0.2$ would be the hypothesis of interest, the following R code should be used:

# Hypothesis of interest

H2 <- "abs(theta_AL) > 0.2; abs(theta_PD) > 0.2"

# Apply GORICA

set.seed(123) # set seed: to obtain the same results when you re-run it

results_H2 <- goric(est, VCOV = VCOV_est, H2,

comparison = "complement", type = "gorica")

results_H2

This renders the following output:

Results:

model  loglik  penalty  gorica  gorica.weights

1          H2   3.912    0.799  -6.226           0.917

2  complement       2.417    1.701  -1.431           0.083

---

The order-restricted hypothesis ‘H2’ has 10.996 times more support

than its complement.

From this, it can be concluded that $H_{2}$ is 11 times more supported than its complement (i.e., any other hypothesis/ordering). Thus, there is convincing support for the hypothesis of interest, which states that the mean difference between surgery and non-surgery is in absolute values larger than 0.2 for both outcomes.

In case $H_{3}:|\theta_{AL}\left|<\right|\theta_{PD}|$ would be the hypothesis of interest, the following code should be used:

# Hypothesis of interest

H3 <- "abs(theta_AL) < abs(theta_PD)"

# Apply GORICA

set.seed(123) # set seed: to obtain the same results when you re-run it

results_H3 <- goric(est, VCOV = VCOV_est, H3,

comparison = "complement", type = "gorica")

results_H3

This renders the following output:

Results:

model  loglik  penalty  gorica  gorica.weights

1          H3   3.912    1.500  -4.824           0.500

2  complement       3.910    1.500  -4.820           0.500

---

The order-restricted hypothesis ‘H3’ has  1.002 times more support

than its complement.

From the output above, it can be concluded that both hypotheses, $H_{3}:|\theta_{AL}\left|<\right|\theta_{PD}|$ and its complement $|\theta_{AL}\left|>\right|\theta_{PD}|$, are equally likely, that is, they have the same support. The maximum log likelihood values are nearly the same and, consequently, the weights largely depend on the penalty values. In that case, the GORICA weights resemble or even equal the penalty weights, the weights based on solely the penalty parts:

library(devtools)

install_github("rebeccakuiper/ICweights")

library(ICweights)

#?IC.weights

#citation("ICweights")

# Weights based on penalty values

IC.weights(2*results_H3$result[,3])$IC.weights

# Note that the penalty is 2*‘penalty’ is 2*results_H3$result[,3]

# This renders penalty weights of:

[1] 0.5 0.5

# which equal the ‘gorica.weights’ above.

Since both hypotheses are of the same size (i.e., have the same penalty), the GORICA weights for the two hypotheses (with approximately the same fit) are also the same. Notably, if the penalty values differed across the hypotheses, the GORICA weights would differ across the hypotheses as well, but the GORICA weights would still equal the penalty weights. When the GORICA weights equal the penalty weights, like here, one can conclude that there is support for the overlap (here, border) of these two hypotheses: $|\theta_{AL}\left|=\right|\theta_{PD}|$, reflecting equal ‘absolute’ strength. Consequently, there is no support for $H_{3}:|\theta_{AL}\left|<\right|\theta_{PD}|$. We do find evidence for ‘$|\theta_{AL}\left|=\right|\theta_{PD}|$’, which can be evaluated in future research.

Hence, the GORICA can evaluate a hypothesis of interest directly and quantify its support (or the lack there of) in comparison with one or more competing hypotheses. This aids in either confirming an a priori theory or in developing a new or competing theory for future research.

## 6. Discussion

This paper demonstrated how theories regarding relationships based on multiple studies can be evaluated using current methods (i.e., null hypothesis testing and AIC) and GORICA. Current methods to test or evaluate hypotheses in meta-analysis can only address equality restrictions and, therefore, do often not address the hypothesis of interest let alone quantify the support for the hypothesis of interest. Fortunately, this is possible, when using the GORICA. Notably, if the goal of the meta-analysis is prediction and not the evaluation of one or more theories/hypotheses, the researcher should not use model selection as I propose in this paper.

I only inspected ‘regular’ meta-analysis accompanied by null hypotheses tests and model selection using the AIC. An increasingly popular meta-analytic method is meta-analytic structural equation modeling (MASEM [23]). This method additionally provides measures of the overall fit of a model, that is, goodness-of-fit indices, which include the AIC. When using MASEM, meta-analyzed estimates can be restricted and, thus, the AIC values of MASEM can be used to evaluate equality-restricted hypotheses regarding effect size parameters. Note that MASEM cannot evaluate restrictions regarding absolute values, like $|\theta_{AL}|$ and $|\theta_{PD}|$ in Sets 2 and 3 in the illustrations section (i.e., Section 5). In that case, one should use $\theta_{AL}$ and $\theta_{PD}$ (or better, $-\theta_{PD}$) instead. When using MASEM, one has to specify and run each of the models separately (including the unconstrained model). MASEM then provides the AIC value for each model, and one then selects the model with the smallest AIC value. To inspect and compare the relative support for these equality-restricted models, one should also inspect the AIC weights, which are not part of MASEM but can be calculated using the ICweights package [22]. MASEM is, thus, like the other current methods only fit for equality restrictions and was therefore not included in the comparison. In case one wants to know more about the similarity of MASEM and the GORICA and/or how the GORICA can be of added value to MASEM, see Appendix D.

To apply the GORICA, a researcher only needs the meta-analyzed estimates of interest and their covariance matrix, which is output in most meta-analytic software. Notably, in the case of a single study where this information is not given (and, thus, not a meta-analysis as discussed in the paper), one should use the original data to apply the GORICA. Alternatively, one can create multiple covariance matrices (based on expertise and previous research) and do a sensitivity/robustness check. By using the GORICA, researchers can quantify the support for their hypothesis/-es of interest. One can, for instance, make claims like: The hypothesis of interest is 10 times more likely than any other theory (i.e., any other expectation about the ordering of parameters); or: The hypothesis of interest $H_{m}$ is 10 times more likely than the competing hypothesis of interest $H_{c}$.

A disadvantage of the GORICA is that it is an asymptotic method: It assumes that the parameters of interest (i.e., the ones used in the hypotheses of interest) are normally distributed (as more methods assume). This may be an unrealistic assumption for some type of parameters and/or for small samples. Nevertheless, simulations thus-far do show that the performance of the GORICA is good (cf. the simulation in Altınışık et al. [3]). An advantage of the GORICA is that—since it only needs the (unconstrained) parameters of interest and their covariance matrix—it can easily be applied to parameters from all types of statistical models. The GORICA can thus also easily be applied to all types of meta-analyzed effect-size estimates, as long as their covariance matrix is also known.

The GORICA is not the only method which can address theory-based hypotheses. There are multiple confirmatory methods: e.g., F-Bar test ([24], pp. 25–4), the Bayes factor (a.o., [25]), and the GORIC [4,5]. The most practical ones, when having secondary data, are the Bayes factor in the R package bain [26] and the GORICA in the R function goric [17] of the restriktor package [18]. The latter is used in this paper, the other methods have, as far as I know, not been applied to meta-analyzed estimates to evaluate theory-based hypotheses. Readers who are more in favor of the Bayesian framework instead of the information-theoretical one are referred to the use of bain. Note that most, if not all, described in this paper also applies to bain. One important difference between the GORICA and Bayesian approaches is that the latter use a prior and, therefore, the results may depend on the choice of the prior, especially when there is one or more equality constraints in one of the hypotheses of interest.

By evaluating theory-based hypotheses using the GORICA, researchers from all types of fields (e.g., psychology, sociology, political science, biomedical science, and medicine) can quantify the support for their hypothesis/-es of interest. Evaluating theory-based hypotheses also increases the statistical power of selecting the correct hypothesis, comparable to one- versus two-sided testing in null-hypothesis testing (cf. [27,28] who show that confirmatory methods evaluating theory-based hypotheses have more power than exploratory ones). Hence, meta-analyses could contribute to theory confirmation and/or development by evaluating a priori specified, theory-based hypotheses. Furthermore, the use of meta-analyzed estimates leads to an increased (combined) sample size, which increases the statistical power as well. The quantification of support and the power increase bolster, for instance, developing evidence-based treatments and policy.

As a final remark, meta-analysis heavily depends on equal or quite similar study designs across the primary studies. If there are differences in designs, either incomparable estimates are aggregated, or one aggregates (via meta-regression) only the estimates of subsets of studies which designs are equal (since the moderator selects studies that are comparable; see Appendix A.1.3 for more details). Instead of aggregating estimates, like in meta-analysis, one could aggregate the support for the hypothesis of interest, as Kuiper et al. [29] do for Bayesian model selection. The next step is to develop such a method for the GORICA.

## Data Availability

The data that illustrates the use of the proposed method are openly available in the metafor R package [9], see also http://www.metafor-project.org/doku.php/analyses:berkey1998 (accessed on 1 June 2022). Additionally, the R scripts for analysing the data are openly available at https://github.com/rebeccakuiper/GORICA_on_MetaAn. (accessed on 1 June 2022).

## Abbreviations

    The following abbreviations are used in this manuscript:

AIC	Akaike’s information criterion
GORIC	generalized order-restricted information criterion
GORICA	generalized order-restricted information criterion approximation
IC	information criterion
MASEM	meta-analytic structural equation modeling
MLE	maximum likelihood estimate

## Appendix A. Hypotheses in Meta-Analysis

Next, I will describe different types of hypotheses that may be of interest in meta-analysis. These hypotheses are often referred to as informative hypotheses [25], inequality-constrained hypotheses, order-restricted hypotheses, or theory-based hypotheses. Such hypotheses are ones that could (or even, should) be of interest (unless one does exploratory research and wants to generate such hypotheses). Subsequently, I describe the hypotheses tested with null hypothesis evaluation, which often, if not always, differ from the theory-based ones.

### Appendix A.1. Hypotheses of Interest in Meta-Analysis

In the next three subsections, I will distinguish four meta-analysis cases based on type of effect size (an effect size measure or model parameter(s)) and type of meta-analysis (meta-analysis or meta-regression). I will exemplify these cases based on hypothetical examples in the context of the empirical studies of Batenburg et al. [30] and Buskens and Raub [31] who study the relationship between buyer–seller trust (ratio measurement level) and past experience between buyer and seller (where the measurement level varies in the cases discussed next). For each of these four cases, I will end with one or more examples of hypotheses that could be of interest to the researcher.

#### Appendix A.1.1. Meta-Analyze an Effect Size Measure

Let us investigate the relationship between the outcome buyer–seller trust (measured at a ratio measurement level) and the predictor past experience between buyer and seller. Let us assume that the predictor past experience is measured by a dummy variable denoting the existence of the buyer–seller relationship, that is, whether they did transactions before. Then, probably, the interest lies in the difference of the mean level of trust between buyer–sellers who have a relationship and those who do not, which can be represented by a Cohen’s *d*. Furthermore, let us assume that there are no differences in the designs of the primary studies: e.g., they all measure the outcome and (grouping) predictor in the same way and they do not include other variables in the model. This case represents the situation where there is an effect size measure (not one or more model parameters) which is aggregated by a meta-analysis (as opposed to aggregation via meta-regression, which would include predictors called moderators to correct for, for instance, study-design differences). Notably, in this type of case, there is often only one estimate that will be aggregated over all primary studies.

The hypotheses of interest often address comparing effect size measures to cut-off/pre-specified values. For instance, in case of Cohen’s *d*, hypotheses of interest can be:

(A1) $$\begin{array}{ccc} H_{s}: & & 0.2<d<0.5\\ H_{m}: & & 0.5<d<0.8\\ H_{l}: & & d>0.8, \end{array}$$

hypothesizing a small and/or medium and/or large effect [32], respectively. In an exploratory setting, one would want to evaluate this set simultaneously; while, in a confirmatory setting, one may only be interested in one of these hypotheses. Note that these cut-off values are arbitrary benchmarks, suggested to be used in the absence of any other information about the size of effects within a particular research field (cf. [33]).

#### Appendix A.1.2. Meta-Analyze Model Parameters

Let us now assume that the predictor past experience is measured by a categorical (ordinal) variable denoting the range of years of existence of the buyer–seller relationship, where for ease three ranges are assumed. Again, assume that there are no differences in the designs of the primary studies. In this situation, there will be three means of trust, namely one for each year-range, that will be aggregated over all primary studies.

As another example, let us assume that the predictor past experience is measured by the worth (in euros) of previous transactions between the buyer and seller. Hence, past experience is now a continuous predictor. Assume that there are two other continuous predictors as well: the worth of expected future transactions and the size of the buyer’s network (measured by the total number of sellers). In this situation, there will be three regression parameters (and an intercept).

These examples reflect the case where (standardized) parameter estimates are aggregated (and not an effect size measure). In such a case, there are often multiple estimates that will be summarized over all primary studies (which may also include estimates of interaction effects). This then results in the (standardized) estimates of the population parameters, for instance, of the population parameters $\theta_{1}$, $\theta_{2}$, and $\theta_{3}$.

The hypotheses of interest often address comparing the (standardized) parameter estimate to each other (see Appendix B.1 for more details on standardizing). In the second example above, one may hypothesize that past experience is a more important predictor for trust than expected future transactions is and that they both have more predictive strength than the network size, which is reflected by $\theta_{1}>\theta_{2}>\theta_{3}$. Two other possible hypotheses of interest are:

(A2) $$\begin{array}{ccc} & & \theta_{1}<\theta_{2}<\theta_{3}\\ & & \theta_{1}>\theta_{2}<\theta_{3}, \end{array}$$

where the first hypothesis states that the third predictor has the highest predictive strength followed by the second predictor and then the first one; and the second hypothesis hypothesizes that the second variable has the smallest predictive strength (without hypothesizing on the relationship between the first and third predictor). One could be interested in only one hypothesis, but one can also be interested in multiple, competing hypotheses (based on various theories/findings from previous literature).

#### Appendix A.1.3. Meta-Regression

In the previous two cases, equal study designs were assumed, which rarely occur because oftentimes there are differences in designs across primary studies such that the resulting (standardized) parameter estimates are not comparable and can thus not be meaningfully summarized. This brings us to the other type of meta-analysis: meta-regression.

Like in the first case (discussed in Appendix A.1.1), let us assume that the predictor past experience is a dummy variable and that the interest lies in Cohen’s *d*. Now, let us additionally assume that there are differences in the designs of the primary studies: e.g., some of the primary studies did correct for transaction characteristics while other did not (or did for only some of them). Then, one may want to condition on this by including (categorical and/or continuous) moderators (i.e., predictors on a study-level) in the meta-analysis model (the third case). Including moderators aids in explaining and reducing the heterogeneity variance in the meta-analysis. Such a model is referred to as meta-regression or (when including a random effect) a mixed-effects meta-analysis, where subgroup analysis is a special case.

Similarly for the second case (discussed in Appendix A.1.2), where, for example, the strength of three (standardized) predictors is compared, there can be differences in study designs: e.g., differences in controlling variables and/or different questionnaires were use to measure the level of trust. Then, one also needs to correct for this by using a meta-regression (the fourth case).

Note that, now, there is not only an estimate for the effect size measure or estimates for the (standardized) parameter estimates, but also one for each moderator. A meta-regression thus also renders (standardized) estimates of the population parameters for the moderators denoted by β; e.g., in case of three moderators: $\beta_{1}$, $\beta_{2}$, and $\beta_{3}$.

The hypotheses of interest can be the same as in the first two cases; but, in the meta-regression cases, one can (also) have expectations regarding the strength/importance of the moderators; e.g.:

$$\begin{array}{ccc} & & \beta_{1}>\beta_{2}>\beta_{3}. \end{array}$$

One can also have expectations regarding a (linear) mix of θs and βs, especially when the moderators are grouping variables. Notably, when the characteristics of the different studies are the focus of the analysis, the meta-analysis is referred to as an exploratory meta-analysis (cf. Anello and Fleiss [34]); see Barker et al. [35] for an example.

Next, I will discuss what types of hypotheses are tested in meta-analysis using classical hypothesis evaluation methods. Unfortunately, they differ from the hypotheses of interest mentioned in this section (i.e., Appendix A.1), but I also tell how one can overcome this problem.

### Appendix A.2. Null Hypotheses

When doing a meta-analysis, one can test or evaluate null hypotheses in which an element of θ or β is set to 0 or a specific value. As an example for meta-analyzing effect size measures, one can test whether the measure equals a specific (cut-off) value:

$$\begin{array}{ccc} H_{0}: & & Cohen^{\prime }s\;d=0.2,\\ H_{u}: & & not\;H_{0}; \end{array}$$

or

$$\begin{array}{ccc} H_{0}: & & Cohen^{\prime }s\;d=0.5,\\ H_{u}: & & not\;H_{0}. \end{array}$$

Instead, one can perform a one-sided test; e.g.:

$$\begin{array}{ccc} H_{0}: & & Cohen^{\prime }s\;d\leq 0.2,\\ H_{u}: & & Cohen^{\prime }s\;d>0.2; \end{array}$$

As an example for meta-analyzing model parameters, one can test, for each parameter, whether it is zero:

$$\begin{array}{ccc} H_{0}: & & \theta_{1}=0,\\ H_{u}: & & not\;H_{0}; \end{array}$$

or whether all (or a selection of them) are zero:

$$\begin{array}{ccc} H_{0}: & & \theta_{1}=\theta_{2}=\theta_{3}=0,\\ H_{u}: & & not\;H_{0}; \end{array}$$

or whether all are equal:

$$\begin{array}{ccc} H_{0}: & & \theta_{1}=\theta_{2}=\theta_{3},\\ H_{u}: & & not\;H_{0}. \end{array}$$

In case of meta-regression, one can (additionally) perform tests on the (standardized) moderators, for instance, whether they have equal importance:

$$\begin{array}{ccc} H_{0}: & & \beta_{1}=\beta_{2}=\beta_{3},\\ H_{u}: & & not\;H_{0}. \end{array}$$

These equality-restricted hypotheses differ from the theory-based hypotheses (exemplified by the hypotheses in Equations (A1) and (A2). In the main text, I describe how one can evaluate theory-based hypotheses directly using the AIC-type inequality-constrained model selection criterion called GORICA.

## Appendix B. Remarks on Specifying Hypotheses

### Appendix B.1. Same Scale

It is important to note that comparing parameters (e.g., $\theta_{1}<\theta_{2}$) is only meaningful if these parameters are measured on the same scale. Therefore, it is sometimes needed to use standardized parameters. Bear in mind that the estimates to be aggregated (θ) already have to be on the same scale because meta-analysis can only meaningfully average comparable estimates. When comparing the predictive strength of continuous moderators (β), one has to standardize these first.

### Appendix B.2. Safeguard Hypothesis

The set of hypotheses of interest should consist of at least two hypotheses. When there are multiple theories, these can be included as competing hypotheses. Let us assume that two competing hypotheses can be found in the literature: $\theta_{1}>\theta_{2},\;\theta_{3}>\theta_{4}$ and $\theta_{1}<\theta_{2},\theta_{3}<\theta_{4}$. These hypotheses do not cover all possible theories (namely, $\theta_{1}<\theta_{2},\;\theta_{3}>\theta_{4}$ and $\theta_{1}>\theta_{2},\;\theta_{3}<\theta_{4}$ are not included). Consequently, when both hypotheses are weak (i.e., when both hypotheses are not supported by the data), GORICA selects the best out of a set of weak hypotheses. Therefore, in case the hypotheses do not cover the whole parameter space (as is also the case when there is only one hypothesis of interest), a safeguard hypothesis should be included [4]. Notably, in the case of, for instance, the hypotheses $\theta_{1}>\theta_{2}$ and $\theta_{1}<\theta_{2}$, they together cover the whole parameter space, then no fail-safe hypothesis is needed.

There are two possible fail-safe hypotheses: (i) the unconstrained hypothesis $H_{u}$, where none of the parameters are restricted, which, therefore, represents all possible theories including the one(s) of interest, and (ii) the complement of the hypothesis/-es of interest, representing all other theories, thus, excluding the one(s) of interest. The unconstrained hypothesis should be used to investigate whether the hypotheses of interest are weak or not, that is, whether they are supported by the data or not. Bear in mind that $H_{m}$ is not weak if $w_{m}>w_{u}$, that is, if $w_{m}/w_{u}>1$. When at least one hypothesis is not weak, the relative support for the hypotheses of interest can be inspected. For instance, if $H_{m}$ is not weak, one can check the relative support of $H_{m}$ and $H_{m^{\prime }}$ via $w_{m}/w_{m^{\prime }}$. Using the complement can be more powerful [36] and acts like another hypothesis of interest. Moreover, when evaluating $H_{m}$ versus its complement $H_{c}$, the interest even lies in $w_{m}/w_{c}$. Notably, in software, the addition of the complement as safeguard is currently only available for one theory-based hypothesis (and, thus, not yet for a set of hypotheses).

### Appendix B.3. Range Restrictions

In the case of range restrictions, there is an upper and lower bound placed on the parameter; e.g., $-0.5<\theta_{1}<0.5$. Such restrictions can be of interest when comparing effect size measures, like Cohen’s *d* or Hedges’ *g*, to pre-defined cut-off values. For example, researchers may hypothesize, based on previous research and/or expertise, that Hegdes’ *g* is medium (0.5) to large (0.8), that is, 0.5<g<0.8, where the bounds in the range restrictions on Hedges’ *g* are based on Cohen [32].

For hypotheses with range restrictions, the fit part of the GORIC and GORICA can be uniquely calculated. The challenge is determining the penalty/complexity part, since the complexity of a hypothesis is only uniquely defined for so-called closed convex cones (and range restrictions are not). When choosing a different scaling of the covariance matrix of the estimates (or using another mean in the null distribution), the penalty value changes. Note that, when using Bayesian model selection, the support for range restrictions depends on the choice of prior. Thus, this range restriction problem applies to both the information-theoretical and Bayesian model selection. Notably, in Bayesian model selection, the results also vary across prior choices when at least one hypothesis has at least one equality restriction, while in information-theoretical model selection (e.g., using GORICA) the results are unique. By using the default calculation in current GORICA software, the penalty for a range restriction comes down to setting the parameters equal to a constant (e.g., $\theta_{1}=0.5$), leading to a penalty of zero, since there is no parameter only a constant (which also holds true then for all choices of scaling of the covariance matrix of the estimates). The logic is that, when looking at the whole space, which is very large, the range restriction is almost a line (hence, an equality) within the whole space. Bear in mind that the log likelihood is calculated under the range restriction, only the penalty is based on the equality then.

### Appendix B.4. Equalities and/or Overlapping Hypotheses of Interest

One should be careful with specifying equality restrictions and/or hypotheses that are subsets or have overlap (as exemplified in Kuiper [20]). Note that equalities are subsets of competing hypotheses where the equalities are replaced by order restrictions (e.g., $\theta_{1}=\theta_{2}$ is a subset of $\theta_{1}<\theta_{2}$, since evaluating the latter is the same as evaluating $\theta_{1}\leq \theta_{2}$ because the probability of finding $\theta_{1}=\theta_{2}$ is 0 (cf. [24])) and that all hypotheses are subsets of the unconstrained hypothesis.

The third set of the Berkey example in Section 5 illustrates the problems one can run into. In that example, it is concluded that $H_{05}:|\theta_{AL}\left|=\right|\theta_{PD}|$ has 0.731/0.269≈2.7 times more support than the unconstrained hypothesis (which includes $H_{05}$). This may not seem to be convincing evidence, but it is. This has to do with evaluating an equality which is almost true in the data, as will be elaborated upon next.

#### Appendix B.4.1. Equalities

In the case of equality restrictions, there is the problem that equalities are hardly ever exactly true. In that case, the data will almost be in agreement with the equality. Then, the maximum log likelihood (fit) value is just a bit lower than the maximum value (i.e., the maximum log likelihood value obtained for the unconstrained hypothesis).

One can circumvent this by stating range restrictions, that is, about-equality restrictions. For instance, θ=0 can be replaced by −0.01<θ<0.01. When looking at a range restriction instead of an equality, the fit will (asymptotically) take on the maximum value when the equality is true. The penalty stays the same as for the equality. So, this improves the performance. However, one needs to carefully think about specifying the (data-driven) ranges. It should not be set too wide, because, then, you obtain maximum fit even when the equality is not true, that is, you may overestimate the fit then.

As an illustration, I will next specify about-equality restrictions for the third set of the Berkey example. Note that such a hypothesis needs to be evaluated with the GORICA, since the AIC can only evaluate equality restrictions. In the R code below, I specified a (data-driven) range based on the standard error of $\theta_{PD}$. I want a small interval around the estimate $\theta_{PD}$: I choose to use −0.1 and 0.1, which reflects a 0.8% confidence interval.

# Set 3

# Specify a (data-driven) range based on the standard error of theta_PD:

# sqrt(diag(VCOV_est)) # se(theta_PD) = 0.04945984

H0_range <- "abs(theta_AL) > abs(theta_PD) - 0.1*0.04945984;

             abs(theta_AL) < abs(theta_PD) + 0.1*0.04945984"

# Apply GORICA

set.seed(123) # set seed: to obtain the same results when you re-run it

results_range <- goric(est, VCOV = VCOV_est, H0_range,

                       type = "gorica")

results_range

# output:

Results:

        model  loglik  penalty  gorica  gorica.weights

1    H0_range   3.912    1.000  -5.823           0.731

2  complement   3.912    2.000  -3.824           0.269

---

The order-restricted hypothesis ‘H0_range’ has  2.718 times more support

than its complement.

In case of evaluating about-equality restrictions which are correct, the log likelihood of that hypothesis will equal the maximum value (here, 3.912), while an equality restriction would lead to a value just lower than the maximum value (here, 3.910, as can be seen in the main text). In this case, the complement also receives (near) maximum fit. Then, the GORICA weights will be solely based on the penalty values (as was also seen in the exampl ein th emain text). This situation is discussed in the next subsection. If the interval was taken wider, then the fit for the complement would be lower, leading to more support for ‘H0_range’.

#### Appendix B.4.2. Overlapping Hypotheses of Interest

In case hypotheses are subsets or when they are not subsets but do overlap, it is possible that some or all hypotheses have the same maximum log likelihood value. In that case, the (ratios of) GORICA weights are solely based on the penalty values. This means that the overlap of the hypotheses for which the maximum log likelihood values are the highest is the best one (which is the smallest subset in the case of subsets). Bear in mind that the support will now be divided among the hypotheses with equal maximum log likelihood values. In case one wants to select the best hypothesis of the set, this is sufficient information. If one wants to (also) obtain the support for the overlap, one should specify a hypothesis denoting the overlap and evaluate that against its complement. Note that the overlap can also be the border of two hypotheses. For example, assume a set comprising the two hypotheses: $\theta_{1}>\theta_{2},\;\theta_{3}$ and $\theta_{1}<\theta_{2},\;\theta_{3}$, where, “$\theta_{3}$” means that $\theta_{3}$ is not restricted, that is, $\theta_{3}$ is freely estimated. If they have the same maximum log likelihood (which leads in this example to equal support because the penalty is also the same in this example), this implies support for the border: $\theta_{1}=\theta_{2},\;\theta_{3}$.

Even though the best hypothesis can be selected, there will be a maximum support based solely on the penalty values of the hypotheses [36]. Namely, when the sample size is large enough, the maximum log likelihood will be the same for both hypotheses which will remain to be the same for increasing sample size (even thought the maximum log likelihood value itself does change). Additionally, the penalty values do not change with sample size (when the sample size is large enough). Thus, the difference in fit remains zero and the difference in penalty remains the same. Consequently, the GORICA weights are then solely based on the sample-size independent penalty values and will, thus, be sample-size independent as well (when the sample size is large enough), leading to a maximum support. Consequently, interpreting the relative support may then not be that meaningful (when not taking this into account).

When evaluating a hypothesis against its complement, it is also possible to obtain nearly equal maximum log likelihood values. In that case, the GORICA weights are based mainly on the penalty values. In that case, the same logic applies: There is support for the overlap of a hypothesis and its complement, hence, their border. The border is reflected by the equality version of the hypothesis of interest. For example, the border of $\theta_{1}>\theta_{2}$ and its complement (i.e., $\theta_{1}<\theta_{2}$) is $\theta_{1}=\theta_{2}$ and that of $\theta_{1}>\theta_{2},\;\theta_{3}>\theta_{4}$ and its complement (consisting of three possible orderings) is $\theta_{1}=\theta_{2},\theta_{3}=\theta_{4}$. Similarly, the overlap of $H_{0}$ and its complement (i.e., the unconstrained), is $H_{0}$ itself.

One can detect support for the overlap/border by inspecting the maximum log likelihood (fit) values. If there are multiple hypotheses with the highest fit value, then there is support for the overlap of these hypotheses. Then, the relative support (ratios of weights) of those hypotheses are based on solely the penalty values (since the fit values are the same). Therefore, it can be insightful to inspect the weights based on the penalties of these hypotheses as well. These can be easily calculated using the R function IC.weights from the package ICweights [22]. This function can transform information criteria values (AIC, BIC, GORIC, GORICA) into information criteria weights but can also be used to determine the weights based on the penalty values (where twice the penalty value should be used, since an information criterion does as well). You can obtain access to the function as follows:

library(devtools) # Make sure you have Rtools

install_github("rebeccakuiper/ICweights")

library(ICweights)

?IC.weights # This also contains examples of how to use the function

citation("ICweights") # In case you use this function, please cite it

#results <- goric(est, VCOV = VCOV_est, H1,

#                 comparison = "complement", type = "gorica")

#IC.weights(2*results$result[,3])$IC.weights # Make sure you use ‘2*’

Below one can find the code to calculate the penalty weights for the about-equality restrictions set of the Berkey example discussed at the end of Appendix B.4.1.

# Weights based on penalty values (rounded using 3 decimals)

round(IC.weights(2*results_range$result[,3])$IC.weights, 3)

# Note that the penalty is 2*‘penalty’ (i.e., 2*results_range$result[,3])

# This renders (rounded) penalty weights of:

 H1  H2

0.731 0.269

# which equal the ‘gorica.weights’ above,

# because the ’loglik’ are the same.

In this example, the weights indeed equal the penalty weights. Hence, it can be said that there is support for the boundary of these hypotheses, which is in this case $H_{0}$ itself.

## Appendix C. GORIC and GORICA

This appendix provides more detail, for those interested, about the log likelihood and penalty parts of the GORIC and GORICA. Most, if not all, information comes from [3].

For ease, assume a univariate normal linear model:

y=Xβ+ϵ,

where $y={\left(y_{1},y_{2},\ldots ,y_{N}\right)}^{T}$ denotes the outcome, $X=(x_{0},x_{1},\ldots ,x_{p},\ldots ,x_{P-1})$ with $x_{p}={\left(x_{p1},x_{p2},\ldots ,x_{pN}\right)}^{T}$ for p=0,1,…,P−1 contains the predictors, $\beta ={\left(\beta_{0},\beta_{1},\ldots ,\beta_{P-1}\right)}^{T}$ are the regression coefficients, and $\epsilon ∼N(0,\sigma^{2}I_{N})$ represents the vector of residuals with mean vector 0 and covariance matrix $\sigma^{2}I_{N}$, where $\sigma^{2}$ is the variance term and $I_{N}$ denotes the N×N identity matrix. For such a model and for hypothesis $H_{m}$, the GORIC is defined as:

$${\mathrm{GORIC}}_{m}=-2\;L\left({\tilde{\beta }}^{m},{\tilde{\sigma }}^{m}|y,X\right)+2\;\left[PT_{m}\left(\beta \right)+PT_{m}\left(\sigma \right)\right],$$

where, ${\tilde{\beta }}^{m}$ and ${\tilde{\sigma }}^{m}$ maximize the log likelihood, L(β,σ|y,X), subject to the restrictions in hypothesis $H_{m}$.

Let us now define the parameters included in the set of hypotheses as structural parameters, denoted by θ (which will contain many if not all βs), and the other parameters as nuisance parameters, denoted by ξ (which will contain many if not all variance terms, here $\sigma^{2}$, and perhaps some βs). As will become clear later, this notation helps in comparing the expressions of the GORIC and the GORICA. Using this notation, the GORIC for hypothesis $H_{m}$ is defined as:

$${\mathrm{GORIC}}_{m}=-2\;L\left({\tilde{\theta }}^{m},{\tilde{\xi }}^{m}|y,X\right)+2\;\left[PT_{m}\left(\theta \right)+PT_{m}\left(\xi \right)\right],$$

where ${\tilde{\theta }}^{m}$ and ${\tilde{\xi }}^{m}$ maximize the log likelihood, L(θ,ξ|y,X), subject to the restrictions in hypothesis $H_{m}$. The order-restricted maximum log likelihood for a univariate normal linear model is:

(A3) $$\begin{array}{cc} L({\tilde{\theta }}^{m},{\tilde{\xi }}^{m}|y,X)= & -\frac{N}{2}\log\left(2\pi \right)-\frac{1}{2}\log\left|{\tilde{\xi }}^{m}I_{N}\right|-\frac{1}{2}\left[{\left(y-X{\tilde{\theta }}^{m}\right)}^{T}{\left({\tilde{\xi }}^{m}I_{N}\right)}^{-1}\left(y-X{\tilde{\theta }}^{m}\right)\right]. \end{array}$$

In the case of solely equality restrictions, the GORIC reduces to the AIC. Thus, in that case, the order-restricted maximum log likelihood equals the maximum log likelihood of the AIC and the penalty of the GORIC equals the number of distinct parameters (as in the penalty of the AIC).

Using the same notation as above, the expression of the GORICA for hypothesis $H_{m}$ is:

$${\mathrm{GORICA}}_{m}=-2\;L\left({\tilde{\theta }}^{m}|\hat{\theta },{\hat{\Sigma }}_{\hat{\theta }}\right)+2\;PT_{m}\left(\theta \right),$$

where ${\tilde{\theta }}^{m}$ is the order-restricted MLE of θ, as we saw before, and $\hat{\theta }$ and ${\hat{\Sigma }}_{\hat{\theta }}$ denote the maximum likelihood estimates of the structural parameters and their covariance matrix, respectively, that are used to construct a normal approximation of the likelihood:

$$L\left({\tilde{\theta }}^{m}|\hat{\theta },{\hat{\Sigma }}_{\hat{\theta }}\right)=-\frac{K}{2}\;\log\left(2\pi \right)-\frac{1}{2}\;\log\left|{\hat{\Sigma }}_{\hat{\theta }}\right|-\frac{1}{2}{\left(\hat{\theta }-{\tilde{\theta }}^{m}\right)}^{T}{\left({\hat{\Sigma }}_{\hat{\theta }}\right)}^{-1}\left(\hat{\theta }-{\tilde{\theta }}^{m}\right).$$

Notably, when the likelihood of the data is unimodal, roughly symmetric and twice differentiable, one can usually accurately approximate it by a normal distribution centered at the MLE (cf. Appendix A in [3]). Therefore, the GORICA uses the order-restricted likelihood of the MLEs (which is a normal) instead of the order-restricted likelihood of data (which does not need to be normal distribution, as is the case, for instance, in a logistic regression model). The unconstrained MLEs are used as a summary for the data, and the log likelihood of the structural MLEs is then maximized under the order restrictions in $H_{m}$. Thus, in the GORICA, $L(\theta |\hat{\theta },{\hat{\Sigma }}_{\hat{\theta }})$ is maximized under the restrictions, which then leads to the order-restricted MLE ${\tilde{\theta }}^{m}$ and the order-restricted maximum likelihood $L({\tilde{\theta }}^{m}|\hat{\theta },{\hat{\Sigma }}_{\hat{\theta }})$.

The expression of the penalty of the GORICA is:

$$PT_{m}\left(\theta \right)=\sum_{l=1}^{K}w_{l}\left(K,{\hat{\Sigma }}_{\hat{\theta }},H_{m}\right)\;l,$$

with *K* the number of structural parameters. The penalty is a function of level probabilities (i.e., $w_{l}$, that is, ${\bar{\chi }}^{2}$ weights; for more details, see [24]) and the number of levels (*l*) which is related to the number of active constraints in a hypothesis. Although there exist closed form expressions for some hypotheses, for many there are none. Therefore, in the goric function [17] of the restriktor package [18], the penalty is (or better, the level probabilities are) calculated using sampling. More information about the calculation of the penalty of the GORICA can be found in Appendix B of [3].

Because sampling is involved, it is wise to specify a seed value, as is done in the main text and in supplementary R files (shared via my GitHub page, accessed on 1 June 2022). When using a seed, the code will render the same results every time it is run (which makes the results reproducible). Moreover, when using a different seed value, one can check the sensitivity of the results to the number of sampling iterations used (if the results are sensitive, then the number of iterations should be increased). Notably, in the case of equality restrictions (e.g., $\beta_{1}=\beta_{2}=\beta_{3}$) or when the restrictions depend on at max one other parameter (e.g., $\beta_{1}>\beta_{2}$ or $\beta_{1}>\beta_{2},\;\beta_{3}$ or $\beta_{1}>0$ or $\beta_{1}>0,\;\beta_{2}>0$), the penalty will not vary for different seed values. Thus, for many (if not all) examples in the main text, there is no sensitivity in the penalty. On the other hand, for restrictions that depend on at least two other parameters (e.g., $\beta_{1}>\beta_{2}>\beta_{3}$), there can be some sensitivity. Therefore, it is good practice to specify a seed value, irrespective of the type of hypotheses. Luckily, the default penalty calculation in the goric() R function is quite robust.

As can be seen from the GORICA expression, one can leave out all the parts related to the nuisance parameters in the GORICA. This is because these parts are constant across hypotheses. Therefore, these parts will cancel out when comparing hypotheses (cf. Appendix A in [3]). Hence, one only needs the estimates of the structural parameters and their covariance matrix. In a meta-analysis, this means that often one only needs this for the fixed effects and not the variance components (e.g., the amount of heterogeneity in a random-effects model).

Asymptotically, the GORIC and GORICA weights will be the same, but the GORIC and GORICA values will not. First, they can differ in penalty term because of the nuisance parameters. Second, they use a different (order-restricted) log likelihood value. Bear in mind that the log likelihood part of the GORICA will not equal the log likelihood of the data, also not in case of solely equality restrictions. Because of both reasons, the GORICA does not reduce to the AIC in case of solely equality restrictions. Nevertheless, the GORICA weights will asymptotically equal the AIC weights. Consequently, when using the GORICA weighs as a proxy for the AIC weights, as done in the illustrations given in the main text, the log likelihood part of the GORICA does not equal the log likelihood of the data; and the penalty of the GORICA also differs from that of the AIC because of the nuisance parameters (which are the variances in the illustrations). As an example, in the main text, for the unconstrained model: (1) The log likelihood based on the structural MLEs is 3.912 (rendered by the GORICA), while the log likelihood of the data is 5.841 (rendered by ‘logLik(metaan)’); and (2) The penalty of the GORICA is 2 because of the two structural parameters, that is, the two fixed effects (i.e., $\theta_{AL}$ and $\theta_{PD}$), while the penalty of the AIC equals 5 (cf. ‘logLik(metaan)’) because of the 3 (error and sampling) variances.

## Appendix D. GORICA vs. AIC in MASEM

MASEM is in a way comparable to the GORICA. MASEM can, for example, use the meta-analyzed effect-size estimates and their covariance matrix to build a SEM model. On this SEM model, one can impose equality restrictions. This then leads to model-fit indices, like the AIC. Similarly, one can evaluate the equality restrictions with the GORICA, which uses the meta-analyzed effect-size estimates and their covariance matrix. Bear in mind that the GORICA can also evaluate hypotheses containing inequality constraints.

MASEM can also use the study-specific effect-size estimates and their covariance matrix to build study-specific SEM models (possibly with equality constraints) and, then, combine the SEM parameters as effect sizes in a meta-analysis. Note that, in this MASEM approach, any equality restriction will already be imposed on the study level. Likewise, the GORICA can be applied to each of the study-specific effect-size estimates and their covariance matrix, which then leads to study-specific GORICA values. Since GORICA values are not effect size estimates and not normally distributed, one cannot meta-analyze them. One should probably take an approach like proposed in Kuiper et al. [29]. How to combine GORICA values is a topic for future research.

Although MASEM is in a way comparable to the GORICA, the GORICA can be an addition to MASEM, like the AIC is. Namely, the GORICA can be applied to the MASEM parameters of interest, (cf. [20], a tutorial that shows how the GORICA can be applied to SEM models). Bear in mind that if MASEM already imposed equality restrictions (say, $\theta_{1}=\theta_{2},\;\theta_{3}$), these have to be part of the hypotheses to be evaluated with the GORICA (say, $\theta_{1}=\theta_{2}>\theta_{3}$). Thus, it may make more sense to apply the GORICA to unconstrained MASEM parameters estimates. Bear in mind that it is also applied to the unconstrained meta-analyzed effect-size estimates. The GORICA can, then, evaluate the hypotheses of interest containing equality and/or inequality constraints; as opposed to the AIC which can only evaluate equality restrictions.
